# Data-Driven Fusion Algorithms for Temperature-Drift Compensation of MEMS Gyroscopes: A Mini Review

**DOI:** 10.3390/mi17080924

**Published:** 2026-07-31

**Authors:** Haoze Lan, Yingjie Xu

**Affiliations:** School of Aerospace Engineering, Xiamen University, Xiamen 361102, China

**Keywords:** MEMS gyroscope, temperature-drift compensation, data-driven fusion algorithm, signal decomposition, deep temporal network

## Abstract

Microelectromechanical systems (MEMS) gyroscopes are now standard rate sensors in inertial navigation, automotive electronics, industrial automation, and medical instrumentation because they are inexpensive, compact, and readily integrated. Their accuracy, however, degrades with temperature: damping and quadrature coupling change, and readout-electronics behavior shifts, producing temperature-dependent zero-rate-output drift, elevated random noise, and poorer long-term stability. Hardware- and structure-based temperature compensation address part of the problem but carry cost and generality penalties, which has moved recent work toward data-driven software-based temperature-drift compensation. This review focuses on the fusion algorithms that have come to dominate that literature, organized as a four-stage pipeline: signal decomposition, learning-based drift modeling, adaptive filtering, and signal reconstruction. We examine how optimizer-tuned variational mode decomposition and improved empirical-mode-decomposition variants separate temperature-related components from noise; how deep temporal networks and optimizer-coupled learners model the nonlinear, time-lagged drift; and how adaptive Kalman variants and time-frequency filtering reconstruct a stable output. We close by identifying four open problems that recur across the recent gyroscope-specific work—cross-device generalization, temperature hysteresis, embedded real-time deployment, and physics-informed lightweight modeling.

## 1. Introduction

Microelectromechanical systems (MEMS) gyroscopes occupy a distinctive niche among inertial sensors: low cost, small footprint, low power, and compatibility with batch silicon fabrication have placed them in consumer devices, vehicles, robots, and increasingly in tactical-grade navigation roles [1,2]. The same miniaturization that makes them attractive also makes their output strongly temperature-dependent. As ambient temperature changes, the structural material properties, the resonator’s mechanical behavior, and the interface-circuit characteristics all shift, and the combined effect appears at the output as temperature-dependent zero-rate-output (ZRO) drift, increased random noise, and degraded long-term output stability [2,3]. For a vibratory gyroscope these thermal effects act through mechanisms that have no direct counterpart in an accelerometer, so temperature-drift compensation for gyroscopes is treated here as a sensor-specific problem rather than a generic inertial-sensor one.

Terminology is used as follows throughout this review. Zero-rate output (ZRO) denotes the gyroscope output when the applied angular rate is zero, whereas ZRO drift refers to the temperature- or time-dependent variation in that output. Temperature compensation is used as a broad term for correcting temperature-dependent changes in ZRO, scale factor, phase, resonant characteristics, or other performance quantities. Temperature-drift compensation refers more specifically to the correction of temperature-induced drift, particularly temperature-dependent ZRO drift. Error compensation is used only as a broader term that may also include nonthermal errors, such as random noise, vibration-induced errors, scale-factor errors, phase errors, and misalignment. Bias instability denotes the low-frequency stochastic fluctuation of the zero-rate bias, commonly quantified using Allan-deviation analysis, and should not be used interchangeably with systematic temperature-dependent ZRO drift.

Temperature-related error-mitigation strategies divide broadly into hardware- and software-based approaches. Hardware-based temperature compensation suppresses thermal error at its source through packaging, oven control, or temperature-stabilized drive and sense electronics. Recent device- and circuit-level studies further indicate that thermal error can also be mitigated through substrate-level thermal design and interface application-specific integrated circuits with on-chip temperature compensation [4,5]. These methods are physically targeted, but they may increase system complexity and calibration burden, especially when device structure, packaging, or operating range changes [6]. Software compensation instead learns a mapping from measurable quantities—temperature, its rate of change, and features of the gyroscope output—to the thermally induced error, then predicts and subtracts it. Because it is implemented after fabrication, software compensation is flexible, inexpensive to update, and portable across hardware revisions, which is why it has become the dominant direction in the recent literature.

Within software-based temperature-drift compensation, a useful organizing distinction is between serial and parallel processing. A serial scheme denoises the raw signal first and then fits a temperature-drift model; it is simple but risks discarding temperature-bearing features during filtering. A parallel scheme first decomposes the raw output into components of differing frequency or physical meaning, then handles noise suppression, drift modeling, and reconstruction on the appropriate components separately, which better preserves the thermal signal while still removing noise [7,8]. Recent work has converged on the parallel view and extended it into multi-algorithm fusion pipelines rather than single models [9]. A representative pipeline couples optimizer-tuned decomposition, a deep temporal predictor, and an adaptive filter with a reconstruction stage; concrete instances combine triangulation-topology aggregation optimizer-based variational mode decomposition (TTAO-VMD) with a one-dimensional convolutional neural network-bidirectional gated recurrent unit-attention network (1D-CNN-Bi-GRU-Attention) and a strong-tracking adaptive Kalman filter (SHAKF) [10], or rime-ice algorithm-optimized variational mode decomposition (RIME-VMD), a particle swarm optimization (PSO)-tuned deep belief network (DBN), and extreme learning machine (ELM) reconstruction [11], or improved complete ensemble empirical mode decomposition with adaptive noise (ICEEMDAN), sample entropy and a multi-objective-optimized ELM [12].

This review concentrates on those data-driven fusion algorithms as applied specifically to MEMS gyroscope temperature-drift compensation, with the emphasis on gyroscope work.

**Figure 1:**
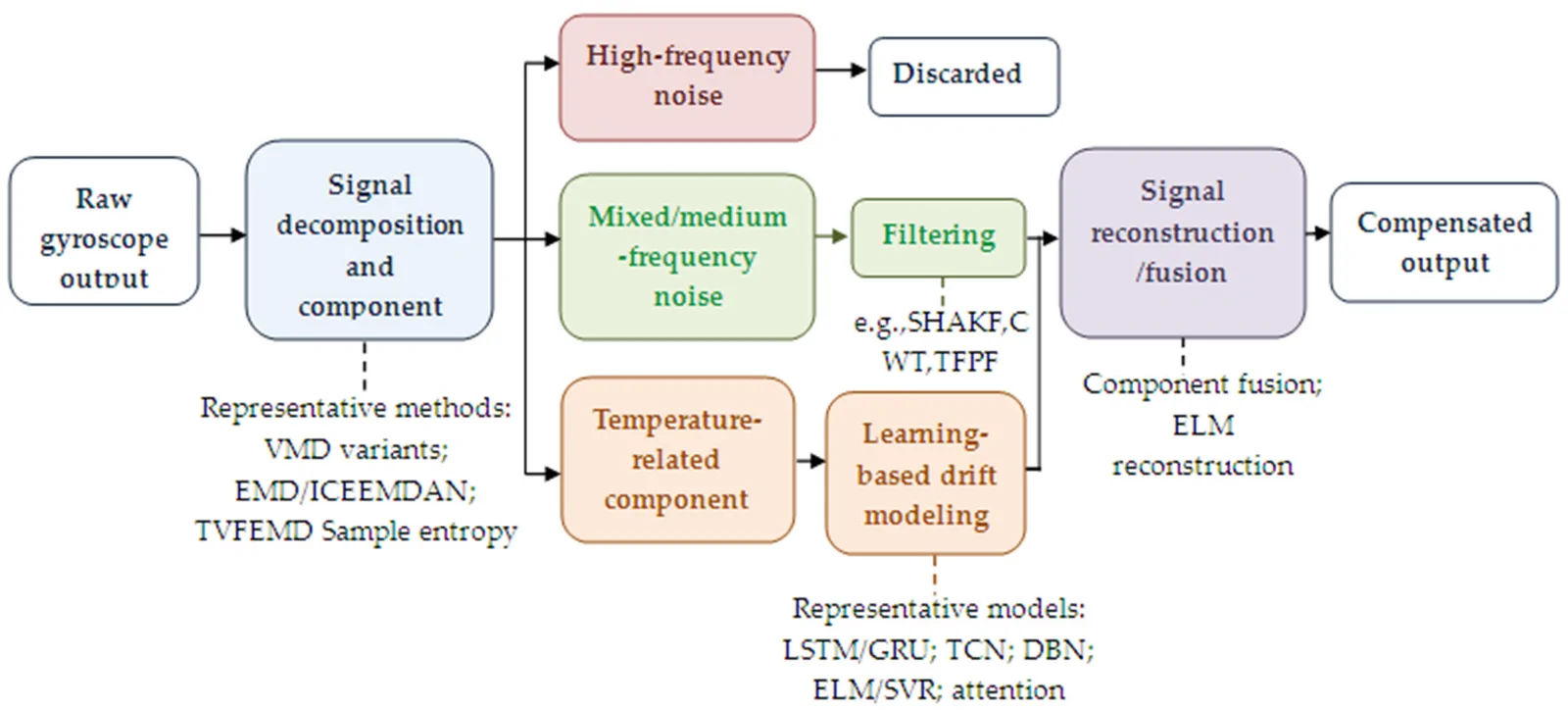
Data-driven fusion pipeline for MEMS gyroscope temperature-drift compensation.

The defining feature of the literature is the move from single temperature-drift compensation models to staged fusion pipelines. The serial-versus-parallel distinction introduced above is the conceptual root: early parallel-processing models for temperature-induced MEMS gyroscope error established that decomposing the output before modeling, rather than denoising it first, preserves temperature-related features that a front-end filter would otherwise remove [7,8]. Recent pipelines build on this by assigning a dedicated algorithm to each processing stage and optimizing the handoff between them. The overall data-driven fusion workflow is illustrated in Figure 1.

## 2. Temperature-Drift Mechanisms

A vibratory MEMS gyroscope senses angular rate through the Coriolis coupling between a driven mode and a sense mode. Temperature perturbs this transduction along several distinct paths. The elastic modulus of silicon falls with rising temperature, shifting the resonant frequencies of both modes and altering the drive-sense frequency split that sets scale factor and sensitivity [2]. Thermoelastic damping and other loss mechanisms change with temperature, modulating the quality factor and therefore the mechanical gain and noise of the resonator; this damping pathway has been characterized directly in MEMS gyroscope structures and is a primary route by which heat couples into bias and stability [3]. Recent physics-oriented studies further show that temperature-induced variations in quality factor, mode matching, substrate stress, and material thermal expansion are closely related to temperature-dependent bias drift, including ZRO drift and scale-factor instability [13,14,15,16,17,18]. Device-level studies under thermo-vacuum conditions further indicate that environmental thermal stability can affect the bias behavior and long-term output stability of MEMS gyroscopes, especially in space-oriented applications [19].

Most temperature-drift compensation pipelines model temperature-dependent ZRO drift directly from temperature inputs and treat residual scale-factor error as a separate, often uncompensated, contributor. In parallel with software compensation, recent high-performance resonator-gyroscope studies have also shown that device-level control strategies can substantially suppress bias instability, although such approaches differ from post-fabrication data-driven compensation [20]. This distinction matters when interpreting the order-of-magnitude bias-instability reductions in Section 5: they are reductions in the additive term under static or quasi-static thermal sweeps, not a full thermal error budget.

The practical consequence is that gyroscope thermal error is nonlinear in temperature, depends on the rate of temperature change as well as its absolute value, and exhibits hysteresis between heating and cooling. The rate dependence is why most recent models take temperature change rate as an explicit input alongside absolute temperature, and the hysteresis is why a model fitted on a heating sweep does not transfer cleanly to cooling.

From a data-driven modeling perspective, these thermal mechanisms suggest a hierarchy of physically meaningful input variables. Absolute temperature T represents the current thermal operating point, whereas the temperature-change rate dT/dt distinguishes heating from cooling and partially reflects transient heat transfer. A finite history of temperature and gyroscope output can represent thermal inertia, delayed structural response, and creep-like behavior. Therefore, data-driven compensation should not be regarded as a purely black-box mapping from temperature to output error; its input design can be guided by the thermal and electromechanical mechanisms summarized above.

## 3. Signal Decomposition for Temperature-Drift Compensation

### 3.1. Representative Signal Decomposition Methods

Signal decomposition functions as the front end of the fusion pipeline. Its purpose is not to compensate for temperature directly but to separate the raw gyroscope output into components so that noise can be removed without sacrificing the temperature-bearing signal that downstream modeling depends on.

The decomposition tools are inherited from general signal processing and are used here only as building blocks. Empirical mode decomposition (EMD) and its ensemble variant (EEMD) adaptively decompose nonstationary signals into intrinsic mode functions but suffer from mode mixing, end effects, and substantial computational cost. Complete-ensemble variants, in particular ICEEMDAN, were introduced to reduce mode mixing and residual noise. Variational mode decomposition (VMD) offers a firmer optimization basis and better noise robustness, but its performance depends on user-set parameters, principally the number of modes and the penalty factor. The gyroscope-specific contribution of recent work is therefore less about the decomposition itself than about removing its manual-tuning dependence and integrating it into the pipeline.

This is where intelligent optimizers enter. A gray wolf optimization-based variational mode decomposition (GWO-VMD) framework uses gray-wolf optimization to set the VMD parameters automatically, reducing the influence of manual selection and retaining the components correlated with valid gyroscope output before passing them downstream [21] (here applied within a multi-frame vibratory gyroscope pipeline). A RIME-VMD framework optimizes VMD with the rime-ice algorithm and partitions a multi-ring disk solid-wave gyroscope output into high-frequency noise, medium-frequency mixed noise, and a temperature-feature component, explicitly supplying the temperature term to the modeling stage [11]. An ICEEMDAN-plus-sample-entropy scheme performs decomposition and component classification together, using sample entropy to sort modes by regularity and mitigate the mode-mixing that limits plain EMD-family methods [12]. The same optimizer-tuned, classification-aware idea extends to the EMD branch: a particle swarm optimization-tuned time-varying filter empirical mode decomposition (PSO-TVFEMD) method for a MEMS ring gyroscope uses particle-swarm optimization to set the TVFEMD parameters, then applies sample entropy to classify the resulting modes and time-frequency peak filtering (TFPF) to denoise them before learning-based modeling [22]. Earlier hybrids established the template: an improved VMD-ELM framework based on a convolutional neural network–long short-term memory network (CNN-LSTM) and particle swarm optimization–support vector machine (PSO-SVM) demonstrated the decomposition-then-learning pattern before the optimizer-tuned variants matured [23].

A point that is easy to lose in the proliferation of optimizer acronyms is that the optimizer does not compensate for temperature; it selects decomposition hyperparameters. Gray-wolf, rime-ice, and triangulation-topology optimizers differ in their search dynamics, but in this pipeline they all solve the same narrow problem of choosing the VMD mode number and penalty factor to minimize a decomposition-quality criterion. The reported performance gains attributed to “GWO-VMD” or “RIME-VMD” are therefore gains from better-conditioned decomposition feeding a stronger downstream model. Across these studies the trajectory is nonetheless consistent: decomposition has shifted from a standalone denoising step into a tunable, classification-aware front end whose output is matched to the needs of the modeling and reconstruction stages that follow.

### 3.2. Comparative Assessment of Signal Decomposition Methods

The decomposition methods reviewed above follow two principal strategies. EMD-family methods perform data-adaptive decomposition according to the local extrema and time-varying characteristics of the signal, whereas VMD formulates decomposition as a global variational optimization problem with a predefined number of band-limited modes. Consequently, EMD-family methods are advantageous when the number and frequency ranges of the signal components are unknown, while VMD generally provides cleaner band separation and greater robustness to mode mixing when suitable parameters are available. TVFEMD occupies an intermediate position by retaining the adaptive nature of EMD while introducing a time-varying filter to improve the separation of closely spaced modes.

As summarized in Table 1, no decomposition method is universally superior. EMD and EEMD provide data-adaptive baselines but are affected by mode mixing and endpoint effects. ICEEMDAN improves decomposition consistency and residual-noise suppression at the cost of repeated ensemble computation. VMD offers a firmer mathematical basis and cleaner band separation, but its performance depends strongly on the mode number and penalty factor. Metaheuristic optimizers alleviate manual parameter selection but introduce an additional offline search cost and stochastic variability. TVFEMD is particularly useful for closely spaced or time-varying components, although its filter-related parameters and computational demand limit embedded implementation. Therefore, the method should be selected according to signal nonstationarity, prior knowledge of the mode structure, available calibration time, and real-time computational constraints.

## 4. Learning-Based Temperature-Drift Modeling

Once the temperature-related component has been isolated, modeling it accurately largely determines the overall temperature-drift compensation accuracy. The recent literature shows a clear progression from shallow regressors toward deep temporal networks and optimizer-coupled learners.

Early data-driven compensation relied on shallow learners, especially support-vector-regression-type models; for example, Nu-support vector regression was used for MEMS error modeling [24]. These models are structurally simple and can generalize from relatively small datasets, but they struggle to represent the full nonlinearity, time dependence, and hysteresis of gyroscope thermal drift.

Deep temporal networks address exactly these properties. Long short-term memory (LSTM) networks, gated recurrent units (GRUs), temporal convolutional networks (TCNs), deep belief networks (DBNs), and attention mechanisms are used to capture long-range temporal dependence and nonlinear temperature features. The recent gyroscope-specific instances combine these into composite predictors. A 1D-CNN-Bi-GRU-Attention model extracts local features with the convolutional layer, captures bidirectional temporal context with the Bi-GRU, and weights the temperature-salient features with attention, taking temperature, temperature rate, time, and a decomposed temperature-related drift component as inputs [10]. A combined TCN-LSTM predictor serves the same role within the GWO-VMD pipeline [21], and a hybrid LSTM-SVM-DBN scheme blends deep and shallow learners to model the drift [25]. Although not all recent learning-based gyroscope calibration studies directly target temperature drift, they provide methodological support for using deep models to handle nonlinear and time-varying gyroscope errors [26,27].

A parallel trend couples learners with intelligent optimizers to stabilize training and improve accuracy. A PSO-tuned DBN uses particle-swarm optimization to set network parameters, mitigating local minima and overfitting and improving the modeling of the temperature-related drift component [11]. A non-dominated sorting genetic algorithm II-optimized extreme learning machine (NSGA-II-ELM) retains the fast training and simple structure of ELM while using multi-objective optimization to improve compensation accuracy and stability jointly [12]. The overall direction is away from single shallow regression and toward fused “deep temporal network plus optimizer” modeling, with the optimizer increasingly responsible for the parameter choices that previously required manual tuning.

The thermal mechanisms discussed in Section 2 provide a physical basis for model-input selection. The recent models do not regress output error on absolute temperature alone; they add temperature change rate, elapsed time, and, in the fusion pipelines, a decomposed temperature-related component as inputs [10,11]. This allows a network to distinguish a warming transient from the same temperature reached while cooling—the information a hysteresis-aware model needs. This input design also exposes a methodological risk that the literature treats unevenly: when training and test segments are drawn from the same continuous thermal sweep, temporal autocorrelation can let a high-capacity temporal network memorize the specific sweep rather than learn a transferable temperature–error relationship. Few studies report a strict separation between training and evaluation temperature cycles, which is one reason the in-sample gains should be read as upper bounds on field performance rather than as generalization estimates. The corresponding physical interpretations and modeling roles are summarized in Table 2.

## 5. Adaptive Filtering and Signal Reconstruction

Modeling does not end the pipeline. The compensated and residual components still need filtering and recombination to yield a stable output, and this final stage is where several recent fusion schemes differentiate themselves.

Conventional Kalman and extended Kalman filters suppress part of the random noise but use fixed process- and measurement-noise parameters; under changing thermal conditions their assumed noise statistics no longer hold, and performance degrades or the filter diverges. Adaptive variants address this by adjusting the noise parameters online. Related virtual–gyroscope fusion work also indicates that improved Sage–Husa adaptive filtering can enhance MEMS gyroscope performance under changing noise conditions [28]. Separate from Kalman filter-based drift suppression, full-temperature-range demodulation phase-error compensation has also been investigated as an in-run correction strategy for MEMS gyroscopes [29]. Recent phase-error and mode-matching studies further clarify how phase delay, force-to-rebalance operation, and mode matching influence scale-factor linearity and bias performance. In particular, real-time phase compensation has been shown to improve scale-factor nonlinearity over temperature variations [30,31,32,33].

A strong-tracking adaptive Kalman filter lineage, developed for gyroscope temperature-drift suppression, is the ancestor of the SHAKF stage used in recent pipelines. In the TTAO-VMD framework, the decomposed output is separated into high-frequency noise, white noise, mixed noise, and a temperature-related component; the high-frequency and white-noise terms are discarded, the mixed-noise term is filtered by SHAKF, and the temperature-related drift component is estimated and corrected by the learning model, after which the cleaned and compensated terms are fused into the final output [10].

The RIME-VMD pipeline reconstructs analogously: it drops the high-frequency term, processes the medium-frequency mixed noise (via continuous wavelet transform (CWT)) and the temperature noise (via PSO-DBN) separately, and recombines through an ELM reconstruction stage [11]. Time-frequency peak filtering provides the reconstruction route in the ICEEMDAN/NSGA-II-ELM scheme [12], and a double-U-beam vibration ring gyroscope has been compensated for with a fusion algorithm targeting real-time temperature compensation and noise suppression jointly [34].

Learning-tuned filtering methods developed for adjacent MEMS gyroscope error sources also provide useful methodological references. For example, a convolutional denoising autoencoder combined with a MultiTCN-Attention Kalman filter has been used for static-base gyroscope noise compensation [35], while an LSTM-plus-Kalman framework has been applied to gyroscope error compensation under random-vibration conditions [36]. Although these studies do not directly address temperature drift, they indicate that filter parameters and residual-error dynamics can be learned from data, which remains insufficiently explored in MEMS gyroscope temperature-drift compensation.

Conventional Kalman filtering is computationally efficient but assumes fixed process- and measurement-noise statistics, making it most suitable for approximately stationary conditions. Sage–Husa adaptive filtering and strong-tracking adaptive Kalman filtering update the noise statistics or state covariance online and are therefore more suitable for temperature-varying and nonstationary conditions. Their performance, however, depends on forgetting factors, fading factors, and covariance-tuning rules, and inappropriate adaptation may cause oscillation or divergence. CWT and TFPF do not rely on an explicit state-space model and are effective for nonstationary residual-noise suppression, but they generally introduce higher computational cost, boundary effects, and processing latency. Learning-assisted filters can adapt residual-error dynamics from data, although their cross-device generalization and stability guarantees remain insufficiently studied. Beyond post-processing filters, recent device- and control-level correction studies, including FTR dual-loop control, modal-frequency tuning, linearized transduction, and parametric modulation, show that in-run control can directly affect ARW, offset drift, and real-time robustness [37,38,39,40].

Angle random walk (ARW) characterizes the growth of short-term angle uncertainty caused primarily by white rate noise and is commonly expressed in °/√h; a lower ARW indicates lower short-term random noise. Bias instability characterizes the minimum level of low-frequency stochastic bias fluctuation, commonly estimated through Allan-deviation analysis and expressed in °/h; a lower value indicates better medium- to long-term zero-rate stability. ARW and bias instability are statistical noise and stability metrics and are therefore distinct from the systematic temperature-dependent variation in ZRO, although effective temperature-drift compensation may reduce all three quantities in the reported experiments. Representative recent data-driven fusion algorithms for MEMS gyroscope temperature-drift compensation are summarized in Table 3.

## 6. Discussion and Future Directions

The studies above demonstrate that staged fusion pipelines can reduce gyroscope thermal-drift metrics by one to two orders of magnitude under their reported test conditions. However, these gains should be interpreted together with the conditions under which they were obtained. The main limitations of the current literature also point directly to the next research directions.

Cross-device generalization and transferable modeling: Most studies train and validate on a single device model, often a single physical unit, over one temperature profile. The headline reductions in Table 3 are therefore largely in-sample with respect to device and protocol, and the cross-device, cross-range behavior of the trained models remains insufficiently characterized [10,11,12,21,25]. Multi-device calibration provides an initial template for the transfer problem [41], but future temperature-compensation models should test transfer across devices, temperature ranges, and repeated thermal cycles rather than only within one continuous sweep.

Temperature hysteresis and standardized benchmarks: Gyroscope thermal error differs between heating and cooling, yet most compensation studies report a single temperature profile and do not separate the two directions. The RIME-VMD study is informative precisely because it reports both: residual angle random walk and bias instability after compensation are higher on the cooling branch than on the heating branch, showing that a model fitted on one direction does not fully capture the other [11]. This limitation is closely tied to the benchmarking problem. A shared MEMS gyroscope thermal dataset with fixed temperature-profile protocols, including separate heating and cooling sweeps, would make it possible to compare algorithms under identical conditions rather than through self-reported in-sample gains. Oven-controlled bias-repeatability characterization can serve as a useful protocol reference [42], but no common benchmark has yet been adopted.

Embedded and real-time deployment: The accuracy gains of TTAO-VMD, RIME-VMD, GWO-VMD, and deep temporal networks come with higher model complexity and computational cost, which conflicts with deployment on the resource-constrained microcontrollers that host most MEMS gyroscopes. Real-time-oriented designs exist, such as the double-U-beam fusion algorithm for simultaneous temperature compensation and noise suppression [34], and compact gyroscope calibration networks show that lightweight inference is feasible in adjacent calibration tasks [43]. Future studies should therefore report latency, memory use, and energy consumption on target hardware, not only reductions in bias instability and angle random walk.

Physics-informed lightweight models as an open direction: Existing mechanism studies provide a basis for physics-guided model design. In addition to temperature, measurable variables such as the quality factor or mode-frequency split may be introduced as model inputs because they reflect temperature-dependent damping and mode matching [13,14,15,16,17,18]. MTEA-type formulations may also provide analytical prior information for thermal-error modeling [44]. A practical research route is therefore to combine a lightweight learner with a small number of physically meaningful variables, rather than relying only on the gyroscope output and ambient temperature.

Constructing a physics-constrained loss function is nevertheless nontrivial. A typical formulation would combine a data-fitting term with one or more physics-residual terms associated with the temperature-dependent quality factor, drive–sense modal-frequency split, or analytical thermal-error relations. However, the coefficients linking these physical quantities to ZRO drift vary with device structure, fabrication variation, packaging stress, and operating range. In addition, quantities such as the quality factor and modal-frequency split may be unavailable or noisy during online operation, while thermal hysteresis and creep are path-dependent and cannot be fully represented by a single static algebraic residual. The relative weighting between the data-fitting and physics-residual terms must also be selected carefully and may not transfer across devices or temperature profiles. Consequently, an overly strong or misspecified physical constraint can introduce systematic bias and reduce compensation accuracy rather than improve it. For these reasons, physics-informed lightweight temperature-drift compensation should currently be regarded as a promising research direction rather than an established general solution.

## 7. Conclusions

Temperature-drift compensation for MEMS gyroscopes has moved decisively from single-model regression toward multi-algorithm fusion pipelines that chain signal decomposition, learning-based drift modeling, adaptive filtering, and reconstruction. Optimizer-tuned VMD and improved EMD-family decompositions now separate temperature-bearing components from noise without manual parameter selection; deep temporal networks and optimizer-coupled learners model the nonlinear, time-lagged drift; and adaptive Kalman variants with time-frequency filtering reconstruct a stable output. The recent gyroscope-specific studies report order-of-magnitude reductions in angle random walk and bias instability, but those gains are largely in-sample and are not yet comparable across studies because metrics and test protocols are unstandardized. Four problems remain genuinely open: cross-device generalization, temperature hysteresis, embedded real-time deployment, and physics-informed lightweight modeling. Progress on the first three is incremental and within reach of current methods; the fourth is, at present, an unfilled direction rather than a demonstrated capability. A compensation method that is simultaneously accurate, low-complexity, generalizable, and physically interpretable is the target the field is converging toward, and a shared benchmark is the precondition for knowing when it has been reached.

## Figures and Tables

**Table 1:** Comparison of representative signal-decomposition methods.

Method	Principle	Strengths	Limitations	Cost	Suitable Use
EMD/EEMD	Local-extrema-based decomposition	Adaptive; no preset basis	Mode mixing; endpoint effects	Low/High	Offline denoising
ICEEMDAN	Ensemble EMD with adaptive noise	Less mode mixing and residual noise	Parameter-sensitive; computationally expensive	High	Complex nonstationary signals
VMD	Variational decomposition into K modes	Clear mode separation; noise-robust	Requires preset K and α	Moderate	Signals with stable frequency bands
Optimized VMD	Metaheuristic tuning of K and α	Less manual tuning; stable decomposition	Offline search cost; stochastic variation	High	High-accuracy offline pipelines
TVFEMD	EMD with a time-varying filter	Separates close and time-varying modes	Parameter-sensitive; relatively slow	Moderate–High	Overlapping nonstationary modes

**Table 2:** Physical basis of candidate inputs for learning-based temperature-drift models.

Model Input	Physical Basis	Modeling Role	Main Limitation
Temperature, T	Thermal operating point	Static drift mapping	No path information
Temperature rate, dT/dt	Thermal transient	Heating/cooling distinction	Noise-sensitive
Temperature/output history	Thermal lag and creep	Memory and hysteresis	Higher latency
Resonant frequency or mode split	Stiffness and mode matching	Structural-state feature	Often unavailable
Quality factor, Q	Temperature-dependent damping	Gain/noise variation	Needs online estimation
Phase or stress indicator	Circuit or package effect	Auxiliary drift state	Extra measurement

**Table 3:** Representative recent data-driven fusion algorithms for MEMS gyroscope temperature-drift compensation. Improvements are reported as stated by the original authors.

Study	Gyroscope	Decomposition/Classification	Drift Modeling	Filtering/Reconstruction	Reported Improvement (As Stated by Authors)
Tan et al., 2026 [10]	Dual-mass vibratory	TTAO-VMD → high-freq, white, mixed, temperature-related	1D-CNN-Bi-GRU-Attention	SHAKF + fusion reconstruction	ARW: 18.56 → 0.17°/$\sqrt{h}$; bias instability: 32.76 → 0.82°/h
Li et al., 2024 [21]	Multi-frame vibratory	GWO-VMD (auto-parameter)	TCN-LSTM	Denoised compensated output	Rate random walk 102.929 → 17.69°/h/$\sqrt{Hz}$ (−82.81%); bias instability 63.70 → 1.38°/h (−97.83%)
Wang et al., 2025 [11]	Multi-ring disk solid-wave	RIME-VMD → high-freq, mid-freq mixed, temperature feature	PSO-DBN	CWT + ELM reconstruction	Heating −40 → 60 °C: bias instability 78.48 → 0.81°/h, ARW 36.20 → 0.15°/$\sqrt{h}$. Cooling 60 → −40 °C: bias instability 49.32 → 0.98°/h, ARW 47.53 → 0.42°/$\sqrt{h}$.
Zhang et al., 2024 [12]	Dual-mass	ICEEMDAN + sample entropy	NSGA-II-ELM	TFPF + reconstruction	Rate random walk 0.531 → 6.66 × 10^−3^°/h/$\sqrt{Hz}$; bias stability: 32.74 → 0.259°/h

Note: ARW, angle random walk; ZRO, zero-rate output. Lower ARW and bias instability values indicate improved gyroscope performance. Because the studies use different devices, temperature profiles, data partitions, and metric definitions, the reported values should not be interpreted as a direct ranking of algorithms.

## Data Availability

No new data were created or analyzed in this review.

## Abbreviations

The following abbreviations are used in this manuscript:

**Abbreviation**	**Definition**
ARW	Angle random walk
CNN	Convolutional neural network
CWT	Continuous wavelet transform
DBN	Deep belief network
ELM	Extreme learning machine
EMD	Empirical mode decomposition
GRU	Gated recurrent unit
ICEEMDAN	Improved complete ensemble empirical mode decomposition with adaptive noise
MEMS	Microelectromechanical systems
PSO	Particle swarm optimization
SHAKF	Strong-tracking adaptive Kalman filter
TCN	Temporal convolutional network
TFPF	Time-frequency peak filtering
VMD	Variational mode decomposition
ZRO	Zero-rate output
